# The Effect of Ketamine, Xylazine, and Ketamine/Xylazine Administration on SCN5A (Nav1.5) Gene Expression in Pigeons

**DOI:** 10.1002/vms3.71062

**Published:** 2026-07-02

**Authors:** Faezeh Sadat Moazzeni, Behnam Pedram, Seyed Morteza Razaghi Manesh

**Affiliations:** ^1^ Department of Veterinary Medicine, Graduate, Sho.C. Islamic Azad University Shoushtar Iran; ^2^ Department of Veterinary Medicine, Sho.C. Islamic Azad University Shoushtar Iran

**Keywords:** anaesthesia, ketamine, pigeon, RT‐qPCR, SCN5A gene, xylazine

## Abstract

**Background Objective:**

Voltage‐gated sodium channels, particularly Nav1.5 encoded by the SCN5A gene, play essential roles in neuronal conduction and cardiac function. Given the widespread use of anaesthetic agents such as ketamine and xylazine in animal studies, this study aimed to evaluate their effects on SCN5A gene expression in pigeon heart tissue.

**Methods:**

A total of 20 healthy male and female pigeons (*Columba livia*) were examined, weighing between 300 and 400 g and aged 6 to 24 months. The birds were randomly divided into four groups of 5, including a control group received normal saline solution (0.9%) at a dose of 1 mL/kg (IM) (group 1), ketamine (60 mg/kg, IM) (group 2), xylazine (16 mg/kg, IM) (group 3), and a combination of ketamine (30 mg/kg, IM) and xylazine (8 mg/kg, IM) (group 4). To study the expression level of the SCN5A gene in pigeon heart tissue, total RNA was first extracted from the tissue samples. Then, the extracted RNA was converted to cDNA, and using quantitative PCR reaction (RT‐qPCR) and specific primers, the expression level of the target gene was measured quantitatively and accurately. The obtained data were analysed and compared using the relative method (ΔCt).

**Results:**

The melting curve analysis revealed a single, sharp peak at approximately 85°C, with no secondary peaks or shoulders, indicating specific amplification and the absence of primer‐dimer or non‐specific products. The primer efficiency was determined using a standard curve generated from serial dilutions of cDNA. The slope was –3.01, and the amplification efficiency was calculated as 98.89%, which falls within the acceptable range (90%–110%), confirming high PCR efficiency and the absence of inhibitors. The statistical analysis showed no significant difference in SCN5A gene expression between the treatment groups.

**Conclusion:**

In conclusion, ketamine, xylazine, and ketamine/xylazine administration did not result in a statistically significant difference in SCN5A expression under the specific experimental conditions tested. Nonetheless, the observed trends warrant further investigation in larger studies or in other target tissues.

## Graphical abstract

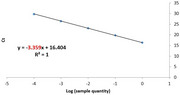

## 1 Introduction

Over the last several decades, significant advancements have been made in the fields of neurology and pharmacology, resulting in substantial improvements in our understanding of the molecular processes that regulate physiological activity in living organisms (Oyebanjo et al. 2024; Zhou and Zhang 2023). In excitable tissues, such as the cardiac myocardium and certain types of nerve cells, voltage‐gated sodium ion channels, particularly the Nav1.5 channel produced by the SCN5A gene, play a crucial role in generating and conducting electrical impulses. This is particularly true for the Nav1.5 channel (Marchal and Remme 2023; Remme 2023; Zhang et al. 2025). Any changes in the expression or function of voltage‐gated sodium ion channels can directly affect the action potential, excitability, and ultimately the proper functioning of these tissues (Savio‐Galimberti et al. 2012). Due to their highly developed and distinctive cardiovascular and neurological systems, birds, particularly pigeons, are valuable models for studying the effects of medications on voltage‐gated sodium ion channel systems.

Some substances used in anaesthesia for laboratory and wild animals include ketamine, a noncompetitive NMDA receptor antagonist, and xylazine, an alpha‐2 adrenergic receptor agonist. Both of these drugs are utilised extensively (Muir 2010; Saha et al. 2005). Ketamine mainly induces a state of dissociative anaesthesia (Kohtala 2021; Nogo et al. 2022; Savić Savić Vujović et al. 2023), whereas xylazine produces sedation, muscle relaxation, and profound analgesia (Ayub et al. 2023; Edinoff et al. 2024; Owusu‐Antwi et al. 2025). In order to provide a better balanced anaesthetic with fewer side effects, it is common practice to utilise a combination of ketamine and xylazine that is administered concurrently (Richardson and Flecknell 2005). Ketamine, a noncompetitive antagonist of the NMDA glutamate receptor, induces dissociative anaesthesia by inhibiting excitatory signal transmission in the central nervous system (Liu et al. 2020; Sleigh et al. 2014). In contrast, xylazine, a potent alpha‐2 adrenergic receptor agonist, produces profound sedative, analgesic, and muscle‐relaxant effects by inhibiting norepinephrine release at synapses (Hoffman et al. 2024). When these two drugs are administered in combination, ketamine modulates the potential stimulatory effects of xylazine on the cardiovascular system (such as initial bradycardia), and xylazine significantly reduces the required ketamine dose and increases anaesthesia stability, ultimately resulting in a more balanced anaesthesia with minimal cardiorespiratory complications.

Although the main mechanisms of action of these drugs are well known, their effects at the molecular level, especially on the expression of key genes such as those encoding ion channels, have been less studied. This lack of information creates an important gap in our comprehensive understanding of the possible long‐term effects of these anaesthetic drugs. The acute administration of these pharmacological agents acts as a stressor, leading to the up‐ or downregulation of important genes, such as SCN5A, which is a significant issue (Imbrici et al. 2016). These alterations may occur even after the medication has been metabolised and eliminated from the body, and they can affect the electrophysiological function of the heart and the nervous system. Reduced expression of SCN5A, for instance, may affect depolarisation and impulse conduction by decreasing the amount of sodium that enters cardiac cells. This may increase a person's susceptibility to cardiac arrhythmias (Li et al. 2018; Remme 2013; Veerman et al. 2015). Therefore, assessing the effects of these drugs at the transcriptional level is an essential step in a thorough evaluation of their safety.

Avian models possess a cardiac conduction system and electrophysiological properties that are distinct from those of mammals (Flores‐Santin and Burggren 2021; Shiels 2022). Studying this species is not only important for its applications in veterinary anaesthesia in birds, but also could provide valuable insights into the evolution and maintenance of ion channel function in poultry. The SCN5A gene in birds also plays a similar role in the heart, and changes in its expression can serve as a sensitive measure of drug‐induced molecular stress (Shiels 2022). However, the available data on the effect of common anaesthetics on the expression of this gene in birds is very limited. Hence, the present study, by designing a controlled experiment, investigates the fundamental question of how single‐dose administration of ketamine, xylazine, and the ketamine–xylazine combination affects SCN5A mRNA expression levels in pigeon heart tissue. The primary objective of this study is to investigate the molecular effects of these common anaesthetics on the expression of a key cardiac gene in pigeons.

## 2 Methods and Materials

### 2.1 Ethical Approval

All applicable international, national, and institutional guidelines for the care and use of animals were followed, and we confirm that the study was conducted in compliance with the ARRIVE guidelines. The ethical committee of the Animal Welfare Committee at Islamic Azad University, Shoushtar branch, has approved the study procedure. All authors have reviewed the ethical issues, including plagiarism, consent to publish, misconduct, data fabrication and falsification, double publication and submission, and redundancy (Ethical code: IR.IAU.AHVAZ.REC.1404.395).

### 2.2 Study Design

A total of 20 healthy male and female pigeons of the *Columba livia* breed were examined, weighing between 300 and 400 g and aged 6 to 24 months. Before treatments, birds were housed for two weeks under constant conditions (temperature, 22–25°C; humidity, 50%–60%; light cycle, 12:12). A diet consisting of mixed grains and fresh water was provided ad libitum. Food and water were removed half an hour before the injection to prevent vomiting.

### 2.3 Grouping

Twenty birds were randomly divided into four groups of 5, including a control group received normal saline solution (0.9%) at a dose of 1 mL/kg (IM) (group 1), ketamine (60 mg/kg, IM) (group 2), xylazine (16 mg/kg, IM) (group 3), and a combination of ketamine (30 mg/kg, IM) and xylazine (8 mg/kg, IM) (group 4). The drugs were injected intramuscularly into the pectoral muscle at a specific time in the morning to minimise circadian rhythm effects. Twenty‐four hours after injection, birds were anaesthetised with ketamine (50 mg/kg) and then euthanised. Immediately after death, left ventricular tissue was removed and frozen in liquid nitrogen, then transferred to a –80°C freezer to prevent RNA degradation.

### 2.4 SCN5A Gene Expression

To study the expression level of the SCN5A gene in pigeon heart tissue, total RNA was first extracted from the tissue samples. Then, the extracted RNA was converted to cDNA. Then, using quantitative PCR (RT‐qPCR) and specific primers, the expression level of the target gene was quantified accurately. The obtained data were analysed and compared using the relative method (ΔCt). A set of specific primers was designed and used to measure the expression level of the SCN5A (Nav1.5) gene as a target gene and a housekeeping gene as an internal control. The primer sequences were designed using gene databases (such as NCBI Primer‐BLAST), and their specific properties, including melting temperature (Tm), amplicon length, and the absence of secondary structures (such as dimers and hairpins), were examined.

#### 2.4.1 Total RNA Extraction

Heart tissues were prepared in a homogenised form using the Trizol reagent or an RNA extraction kit (Qiagen RNeasy). The quality and quantity of the extracted RNA were measured using a spectrophotometer (NanoDrop), and its quality was confirmed by 1% agarose gel electrophoresis.

#### 2.4.2 cDNA Synthesis

When synthesising complementary DNA (cDNA), high‐quality RNA was used with a Reverse Transcription Kit, and the manufacturer's directions were strictly followed. Through this process, RNA is converted into cDNA, which serves as the template for quantitative polymerase chain reaction (qPCR) amplification.

#### 2.4.3 Primer Design

SCN5A gene‐specific primers (NaV1.5) were designed using OLIGO7 software and prepared by Pishgam Synthes Company. These primers are used to amplify target cDNA segments specifically. The specific primers for the target gene (SCN5A) and the reference gene (GAPDH) in *Columba livia domestica* were designed based on their respective GeneBank accession numbers (XM_005513262.3 and NM_021544). The forward (F: TGATGACATCAGAAGGTGGTG) and reverse (R: GTGGAGTCATACTGGAACATG TAG) primers for SCN5A yield an amplicon length of 130 bp. In comparison, the primers for GAPDH (F: CAGACACTGATGACCAGGAAGAG and R: TCGGCTTCAGAGGATGTGGTCT) produce a 150 bp product. The reference gene (GAPDH) was selected due to its stable expression across the studied tissues and under various experimental conditions.

#### 2.4.4 Quantitative PCR (RT‐qPCR)

The expression of the SCN5A gene was determined using a Real‐Time PCR (RT‐qPCR) instrument. SYBR Green kits or particular probes were used in order to carry out the RT‐qPCR experiment. The gadget autonomously managed the thermocycling process settings, including annealing, denaturation, and extension phases.

#### 2.4.5 RT‐qPCR Data Analysis

RT‐qPCR data were analysed using the threshold cycle (ΔCt) method, and the expression level of the SCN5A gene in the experimental groups was calculated relative to the control group. Reference genes (Housekeeping genes) such as GAPDH or β‐actin were used for normalisation.

### 2.5 Statistical Analysis

Data were analysed using SPSS 26. One‐way analysis of variance (ANOVA) was used to compare SCN5A expression levels (ΔCt values) among the four groups. Tukey's post hoc test was used when a statistically significant difference was found. To determine whether the data distribution was normal, the Shapiro–Wilk test was used. If any of these tests were deemed essential, a logarithmic transformation or nonparametric tests, such as the Kruskal–Wallis test, were used. The threshold of significance was set at *p* < 0.05.

## 3 Results

### 3.1 Melting Curve and Primer Efficiency

**FIGURE 1:**
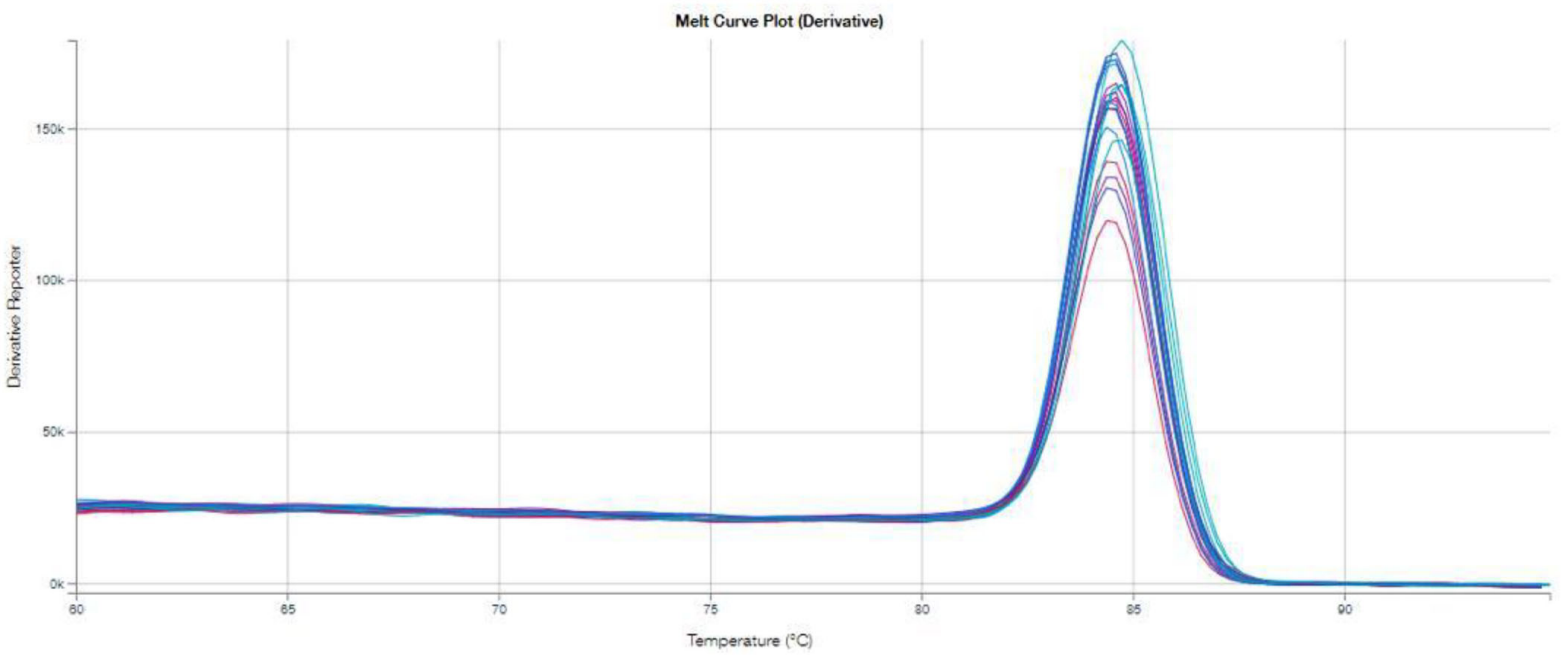
Melting curve of SCN5A gene primers in RT‐qPCR reaction. This curve indicates the uniformity and specificity of PCR product amplification. A single and distinct peak indicates the presence of a single specific product and the absence of non‐specific products or primer dimers. Melting curve analysis was performed using the device software.

**FIGURE 2:**
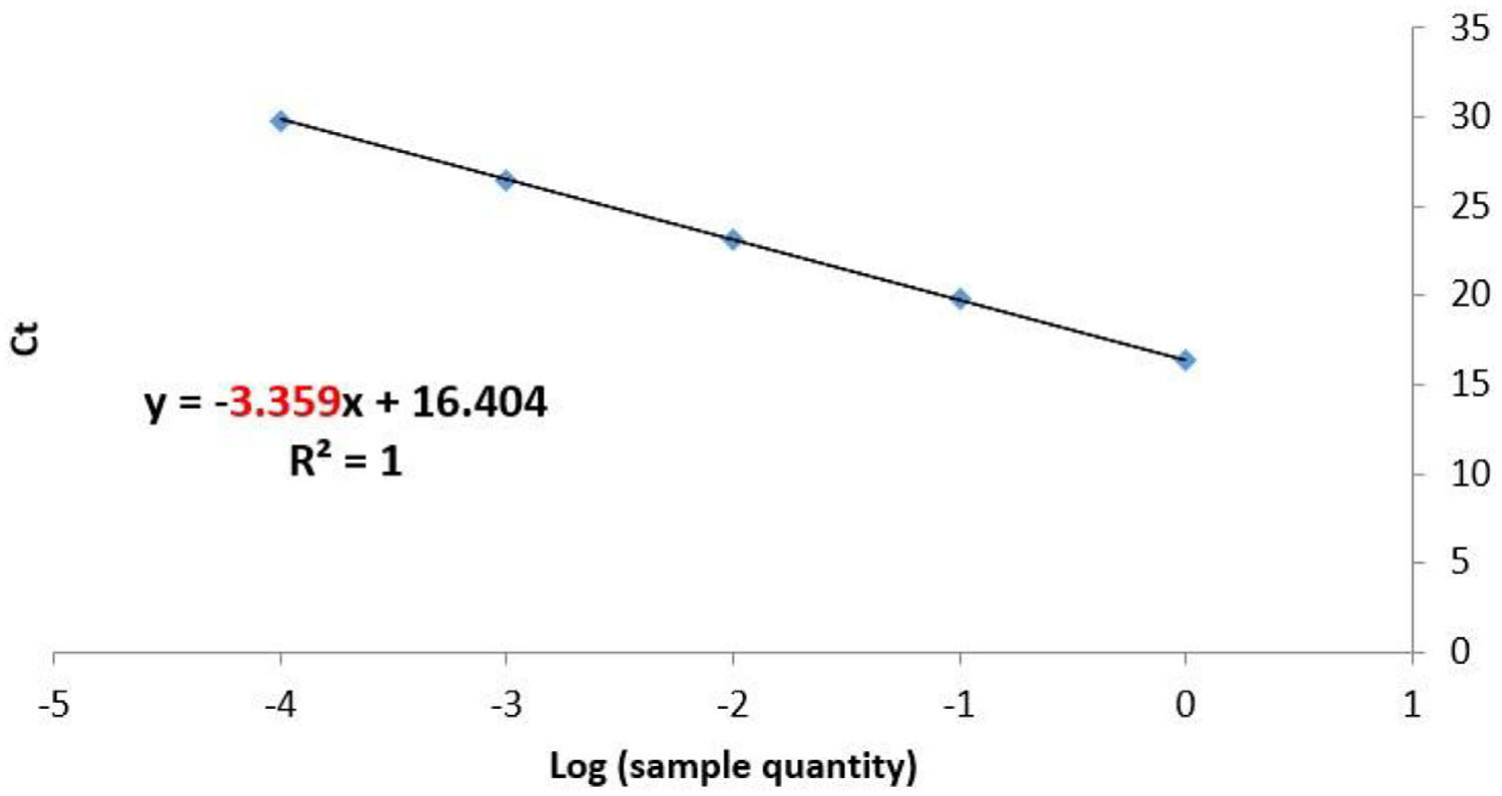
Efficiency plot of SCN5A gene primers in RT‐qPCR reaction. This plot was drawn based on serial dilutions of the cDNA sample, and the Ct value was recorded at each dilution. The slope was approximately –3.01, and the efficiency was 98.89%, indicating the appropriate efficiency and high accuracy of the primers for target amplification under these experimental conditions.

The melting curve analysis revealed a single, sharp peak at approximately 85°C, with no secondary peaks or shoulders, indicating specific amplification and the absence of primer‐dimer or non‐specific products (Figure 1). The primer efficiency was determined using a standard curve generated from serial dilutions of cDNA. The slope was –3.01, and the amplification efficiency was calculated as 98.89%, which falls within the acceptable range (90%–110%), confirming high PCR efficiency and the absence of inhibitors (Figure 2).

### 3.2 Statistical Analysis of SCN5A Gene Expression

To compare SCN5A gene expression levels (ΔCt) among the four groups (control, xylazine, ketamine, and ketamine/xylazine combination), a one‐way analysis of variance (ANOVA) was performed after confirming normality (Shapiro–Wilk test, *p* > 0.05) and homogeneity of variances (Levene's test, *p* > 0.05). The ANOVA showed no statistically significant difference among the four groups. These results indicate that under the specific experimental conditions tested, no statistically significant differences in SCN5A gene expression were detected among any of the treatment groups (Table 1).

**TABLE 1:** Pairwise comparison of SCN5A gene expression (ΔCt) among treatment groups.

Comparison group (A)	Comparison group (B)	Mean difference (A – B)	95% CI for mean difference	*p*‐value
Control	Xylazine	−0.08	(–1.23 to 1.07)	0.892
Control	Ketamine	0.20	(–0.95 to 1.35)	0.678
Control	Ketamine/xylazine	0.00	(–1.15 to 1.15)	0.945
Xylazine	Ketamine	0.08	(–1.12 to 1.28)	0.761
Xylazine	Ketamine/xylazine	−0.12	(–1.32 to 1.08)	0.838
Ketamine	Ketamine/xylazine	−0.10	(–1.20 to 1.20)	0.924

## 4 Discussion

In this study, the relative expression level of the SCN5A gene in heart tissue from pigeons treated with ketamine, xylazine, or a combination of the two showed no statistically significant difference between groups. This finding suggests that the anaesthetic drugs used in this study, at the applied doses, do not significantly affect the expression of the SCN5A gene in pigeon hearts. The current study indicates the relative stability of SCN5A gene's expression in response to the acute effects of anaesthesia, or suggests its insensitivity to the molecular pathways activated by the drugs in question.

In line with the present study, a study by Aizawa et al. (2025) shows that the type of SCN5A gene variants, especially non‐missense variants that cause loss of Nav1.5 sodium channel function, is significantly associated with an increased risk of fatal arrhythmic events in patients with Brugada syndrome (Aizawa et al. 2025). This finding highlights the significance of mutation type and its impact on protein function. In contrast, the present study focused on examining the expression level of the SCN5A gene in the hearts of pigeons treated with ketamine, xylazine, and their combination. The results showed that these drugs had no significant effect on the relative expression of this gene. Thus, although both studies relate to SCN5A, our findings suggest that the functional changes in the sodium channel caused by gene variants may be independent of changes in gene expression. In other words, the findings of this research indicate that the effects of anaesthetics on SCN5A are likely mediated by mechanisms other than gene expression regulation. These mechanisms may include alterations in protein structure or the functioning of sodium channels. It is essential to conduct more comprehensive molecular and functional research in the future to gain a deeper understanding of how gene variations and environmental factors, such as anaesthetics, affect Nav1.5 function and its associated clinical consequences. This significant disagreement between the two studies underscores the need to undertake comprehensive molecular and functional investigations.

In addition to investigating the effects of anaesthetics on physiological parameters, Panprom et al. (2024) evaluated the effects of four different anaesthetic protocols on heart rate variability (HRV), cardiac function, and blood pressure in cats. It was found that ketamine use increased the standard deviation of sinus intervals (SDNN) and decreased the percentage of partial ventricular shortening (FS), suggesting an adverse effect on cardiac function. These findings are consistent with some of the results of the present study; since, despite the lack of significant changes in the expression level of the SCN5A gene, ketamine may be effective in changing the electrophysiological function of the heart through non‐genetic or indirect effects on the function of ion channels, including Nav1.5 (the product of the SCN5A gene).

In line with the current study, Zhou et al. (2023) identified a novel frameshift mutation in the SCN5A gene that caused idiopathic ventricular fibrillation (IVF) with J waves in the inferior leads and increased S wave rise time in the precordial leads (Zhou et al. 2023). Similar to our findings that changes in SCN5A gene expression under ketamine and xylazine anaesthesia were not significantly different, this study also showed that this frameshift mutation causes a ‘loss of function’ of the cardiac sodium channel through a haploinsufficiency mechanism; so that in laboratory cells containing the mutation, sodium current was not recorded even though the defective channel protein was present in the cells. This reduction in sodium channel function delays cardiac electrical conduction, which may manifest as electrocardiographic signs such as J waves and increased S‐wave rise time. Therefore, the results of this study are consistent with our previous findings, which indicate that functional changes in SCN5A, although observed in different models, can affect electrical activity and possibly gene expression. However, under anaesthesia with ketamine and xylazine, the expression level of the SCN5A gene in the pigeon heart does not show significant changes. This may indicate tissue differences or the complexity of regulating this gene's expression across different physiological and pathological conditions.

According to Plumereau et al. (2021), the type of SCN5A variants can play a significant role in predicting the risk of serious arrhythmia complications in patients with Brugada syndrome. The results of their study showed that non‐missense variants are associated with a higher risk of fatal arrhythmia events. However, after diagnosis, no significant difference was observed between the variant types. These findings are somewhat consistent with the results of the present study, which examined SCN5A gene expression under various drug conditions, and emphasise the importance of considering variant type in both molecular and clinical analyses.

Feng et al. (2013) showed that the combination of ketamine and xylazine in severely burned rats led to a significant decrease in cardiac function parameters such as shortening fraction (FS%) and EF, increased serum troponin I levels, and intensified myocardial apoptosis. According to these findings, the combination in question has a damaging impact when subjected to very high levels of physiological stress. However, the average expression of this gene was higher in the group that received the combination of ketamine and xylazine than in the individual groups, suggesting the activation of compensatory mechanisms or feedback regulation. This was observed in the present study, despite the absence of a significant change in SCN5A gene expression in pigeon hearts. Based on this study, it appears that the species, the animal's physiological condition, and the tissue being researched may have a substantial impact on the outcomes of anaesthetic medications.

Moreover, Balla et al. (2021) investigated the function of two novel mutations in the SCN5A gene, which is associated with Brugada syndrome. These mutations result in reduced function of the cardiac sodium channel Nav1.5, leading to varying degrees of loss of function and severe clinical symptoms and arrhythmias in patients. These findings differ from the results of the present study, which showed no significant change in SCN5A gene expression under the influence of the drugs ketamine and xylazine. The current study focused more on changes in gene expression than on the function of the protein channels. However, both studies emphasise that a detailed investigation of the molecular and functional effects of the SCN5A gene is necessary to understand clinical outcomes better, and that multiple factors, such as mutation type and environmental or drug conditions, may play a role.

Furthermore, Cuijpers et al. (2020) demonstrated that the use of the ketamine/xylazine combination, compared with isoflurane, enables more effective detection of diastolic dysfunction in an animal model of HFpEF using echocardiography. This advantage was partially attributed to a decrease in heart rate and to better Doppler wave resolution. The findings of this study indicate that the choice of anaesthesia type can directly affect the quality and accuracy of cardiac assessments in vitro. In the present study, although the focus was on SCN5A gene expression in the heart of pigeons and no significant difference was observed between drug groups, the relative increase in the mean expression of this gene in the ketamine/xylazine combination group could be an indication of the potential effect of these drugs on electrophysiological regulatory pathways or compensatory mechanisms.

Additionally, Matsubara and da Silva‐Santos (2024) showed that the central effects of clonidine on blood pressure and heart rate differed depending on the type of anaesthesia; in rats anaesthetised with pentobarbital, central injection of clonidine resulted in a decrease in blood pressure and bradycardia, while in rats anaesthetised with ketamine/xylazine, clonidine produced a weaker hypertensive and bradycardia effect. These findings suggest that the ketamine/xylazine combination may stimulate central alpha2‐adrenergic receptors, resulting in a distinct cardiovascular response pattern compared to other anaesthetics. This could also be important in interpreting the relative, albeit nonsignificant, increase in SCN5A gene expression in the combination group in the present study; because this drug combination probably exerted effects on the expression of genes related to sodium channels at the cardiac level through central nervous pathways or indirect modulation of cardiac electrical pathways, Matsubara et al.'s findings could help to understand better the consequences of choosing the type of anaesthesia in molecular studies.

### 4.1 Challenges and Future Prospects

Despite the careful and controlled design of the present study, this study faced limitations such as a relatively small sample size (20 pigeons) that may have reduced statistical power, focusing solely on the SCN5A gene in heart tissue that does not provide a complete picture, sampling only at a time point immediately after anaesthesia, and being limited to a specific species (domestic pigeons) that limits the generalisability of the results. Accordingly, it is suggested for future studies to increase the sample size, examine SCN5A gene expression in other tissues such as the heart or brainstem, evaluate gene expression changes at different time points after drug injection, study the effects of different drug doses on other ion channel genes, conduct comparative studies between different species, and simultaneously examine the expression levels of related proteins to establish a more precise link between molecular and physiological changes.

Moreover, another limitation of the present study was examining SCN5A gene expression at the mRNA level using RT‐qPCR, and lacked confirmation at the protein level (such as Western blot), and also the lack of functional assessments, such as electrophysiological measurements or ECG. Given that the main function of the cardiac sodium channel is dependent on the resulting protein and its electrical activity, further studies at the protein and functional levels seem necessary to more fully understand the role of SCN5A in the conditions under study. Another limitation of the present study is that gene expression was assessed at only one time point (24 h after intervention). This study design is unable to identify transcriptional changes at early or late stages. Future studies using a time‐course analysis including multiple time points (e.g., 6, 12, 24, 48 h, or more) could provide a more comprehensive understanding of the temporal pattern of SCN5A expression and significantly strengthen the present findings.

Another limitation of the present study was the use of only one housekeeping gene (GAPDH) to normalise RT‐qPCR data without examining the stability of its expression under the experimental conditions studied. Ideally, at least two stable reference genes should be used for more accurate normalisation. Therefore, it is recommended that in future studies, in addition to GAPDH, other housekeeping genes such as β‐actin, 18S rRNA, or B2M should be used, and their expression stability should be evaluated under similar conditions to increase the accuracy and reliability of the results obtained from gene expression analysis.

Another limitation of the present study was the use of re‐anaesthetisation with ketamine before euthanasia of the animals. Ketamine as an anaesthetic may affect the expression of some genes, including genes involved in cell signalling pathways, and is considered a potential confounding factor. Although the use of anaesthesia was necessary for ethical reasons and to reduce animal pain and suffering, its possible impact on gene expression results cannot be ignored. It is recommended that alternative anaesthesia protocols or appropriate control groups be used in future studies to isolate the effect of anaesthetics on SCN5A expression.

## 5 Conclusion

In conclusion, ketamine, xylazine, and ketamine/xylazine administration did not result in a statistically significant difference in SCN5A expression under the specific experimental conditions tested. This finding suggests that, although these drugs have known effects on the nervous system and neuronal electrical activity, they do not cause a noticeable change in the transcriptional regulation of the SCN5A gene. One possible interpretation is that the regulation of this gene's expression is influenced by more complex factors, such as tissue type, duration of drug exposure, or higher doses, or that the effects of these drugs are more pronounced at the level of channel function (protein function) rather than at the level of gene expression. It is also possible that gene responses to anaesthesia in birds differ from those in mammals and require further investigation.

## Author Contributions

Conceptualisation: B.P. Methodology: F.S.M., B.P., S.M.R.M. Formal analysis and investigation: F.S.M., B.P., S.M.R.M. Writing – original draft preparation: F.S.M., B.P., S.M.R.M. Writing – review and editing: F.S.M., B.P., S.M.R.M. Funding acquisition: B.P. Supervision: B.P. All authors checked and approved the final version of the manuscript for publication in the present journal.

## Funding

The authors have nothing to report.

## Ethics Statement

All applicable international, national, and institutional guidelines for the care and use of animals were followed, and we confirm that the study was conducted in compliance with the ARRIVE guidelines. The ethical committee of the Animal Welfare Committee at Islamic Azad University, Shoushtar branch, has approved the study procedure. All authors have reviewed the ethical issues, including plagiarism, consent to publish, misconduct, data fabrication and falsification, double publication and submission, and redundancy (Ethical code: IR.IAU.AHVAZ.REC.1404.395).

## Conflicts of Interest

The authors declare that they have no conflict of interest.

## Data Availability

The datasets generated during and/or analysed during the current study are available from the corresponding author upon reasonable request.

## References

[vms371062-bib-0001] Aizawa, T. , T. Makiyama , H. Huang , et al. 2025. “SCN5A Variant Type‐Dependent Risk Prediction in Brugada Syndrome.” Europace 27, no. 2: euaf024. 10.1093/europace/euaf024. 39931825 PMC11844247

[vms371062-bib-0002] Ayub, S. , S. Parnia , K. Poddar , et al. 2023. “Xylazine in the Opioid Epidemic: A Systematic Review of Case Reports and Clinical Implications.” Cureus 15, no. 3: e36864. 10.7759/cureus.36864. 37009344 PMC10063250

[vms371062-bib-0003] Balla, C. , E. Conte , R. Selvatici , et al. 2021. “Functional Characterization of Two Novel Mutations in SCN5A Associated With Brugada Syndrome Identified in Italian Patients.” International Journal of Molecular Sciences 22, no. 12: 6513. 10.3390/ijms22126513. 34204499 PMC8234720

[vms371062-bib-0004] Cuijpers, I. , P. Carai , P. Mendes‐Ferreira , et al. 2020. “The Effect of Different Anaesthetics on Echocardiographic Evaluation of Diastolic Dysfunction in a Heart Failure With Preserved Ejection Fraction Model.” Scientific Reports 10, no. 1: 15701. 10.1038/s41598-020-72924-5. 32973263 PMC7518268

[vms371062-bib-0005] Edinoff, A. N. , S. Sall , W. C. Upshaw , et al. 2024. “Xylazine: A Drug Adulterant of Clinical Concern.” Current Pain and Headache Reports 28, no. 5: 417–426. 10.1007/s11916-024-01211-z. 38507135 PMC11126434

[vms371062-bib-0006] Feng, Y. , J. Chai , W. Chu , L. Ma , P. Zhang , and H. Duan . 2013. “Combination of Ketamine and Xylazine Exacerbates Cardiac Dysfunction in Severely Scalded Rats During the Shock Stage.” Experimental and Therapeutic Medicine 6, no. 3: 641–648. 10.3892/etm.2013.1213. 24137240 PMC3786838

[vms371062-bib-0007] Flores‐Santin, J. , and W. W. Burggren . 2021. “Beyond the Chicken: Alternative Avian Models for Developmental Physiological Research.” Frontiers in Physiology 12: 712633. 10.3389/fphys.2021.712633. 34744759 PMC8566884

[vms371062-bib-0008] Hoffman, G. R. , C. Giduturi , N. J. Cordaro , et al. 2024. “Classics in Chemical Neuroscience: Xylazine.” ACS Chemical Neuroscience 15, no. 11: 2091–2098. 10.1021/acschemneuro.4c00172. 38747710

[vms371062-bib-0009] Imbrici, P. , A. Liantonio , G. M. Camerino , et al. 2016. “Therapeutic Approaches to Genetic ion Channelopathies and Perspectives in Drug Discovery.” Frontiers in Pharmacology 7: 121. 10.3389/fphar.2016.00121. 27242528 PMC4861771

[vms371062-bib-0010] Kohtala, S. 2021. “Ketamine‐50 Years in Use: From Anesthesia to Rapid Antidepressant Effects and Neurobiological Mechanisms.” Pharmacological Reports 73, no. 2: 323–345. 10.1007/s43440-021-00232-4. 33609274 PMC7994242

[vms371062-bib-0011] Li, W. , L. Yin , C. Shen , K. Hu , J. Ge , and A. Sun . 2018. “SCN5A Variants: Association With Cardiac Disorders.” Frontiers in Physiology 9: 1372. 10.3389/fphys.2018.01372. 30364184 PMC6191725

[vms371062-bib-0012] Liu, G.‐l. , Y.‐f. Cui , C. Lu , and P. Zhao . 2020. “Ketamine a Dissociative Anesthetic: Neurobiology and Biomolecular Exploration in Depression.” Chemico‐Biological Interactions 319: 109006. 10.1016/j.cbi.2020.109006. 32084352

[vms371062-bib-0013] Marchal, G. A. , and C. A. Remme . 2023. “Subcellular Diversity of Nav1. 5 in Cardiomyocytes: Distinct Functions, Mechanisms and Targets.” The Journal of Physiology 601, no. 5: 941–960. 10.1113/JP283086. 36469003

[vms371062-bib-0014] Matsubara, N. K. , and J. E. da Silva‐Santos . 2024. “The Dual Cardiovascular Effect of Centrally Administered Clonidine: A Comparative Study Between Pentobarbital‐ and Ketamine/Xylazine‐Anesthetized Rats.” Future Pharmacology 4, no. 1: 17–29. 10.3390/futurepharmacol4010003.

[vms371062-bib-0015] Muir, W. W. 2010. “NMDA Receptor Antagonists and Pain: Ketamine.” Veterinary Clinics: Equine Practice 26, no. 3: 565–578. 10.1016/j.cveq.2010.07.009. 21056300

[vms371062-bib-0016] Nogo, D. , H. Nazal , Y. Song , et al. 2022. “A Review of Potential Neuropathological Changes Associated With Ketamine.” Expert Opinion on Drug Safety 21, no. 6: 813–831. 10.1080/14740338.2022.2071867. 35502632

[vms371062-bib-0017] Owusu‐Antwi, P. , P. Atodaria , E. Appiah‐Kubi , Z. Shah , and E. M. Garcia . 2025. “Management of Xylazine Toxicity, Overdose, Dependence, and Withdrawal: A Systematic Review.” The American Journal on Addictions 34, no. 6: 589–602. 10.1111/ajad.70051. 40476542 PMC12555106

[vms371062-bib-0018] Oyebanjo, O. T. , B. O. Adetuyi , A. D. Adeoye , O. A. Adetuyi , P. G. Oni , and O. O. Ogunlana . 2024. “Neuropharmacology and Neurotherapeutics: Advancing the Understanding and Treatment of Neurological Disorders.” In Biochemical and Molecular Pharmacology in Drug Discovery, 403–425. Elsevier. 10.1016/B978-0-443-16013-4.00019-1.

[vms371062-bib-0019] Panprom, C. , N. Pattanapon , and S. Petchdee . 2024. “The Effects of Anesthetic Drug Choice on Heart Rate Variability and Echocardiography Parameters in Cats.” Scientific Reports 14, no. 1: 316. 10.1038/s41598-024-51162-z. 38172353 PMC10764780

[vms371062-bib-0020] Plumereau, Q. , O. Theriault , V. Pouliot , et al. 2021. “Novel G1481V and Q1491H SCN5A Mutations Linked to Long QT Syndrome Destabilize the Nav1. 5 Inactivation State.” CJC Open 3, no. 3: 256–266. 10.1016/j.cjco.2020.09.023. 33778442 PMC7984979

[vms371062-bib-0021] Remme, C. A. 2013. “Cardiac Sodium Channelopathy Associated With SCN5A Mutations: Electrophysiological, Molecular and Genetic Aspects.” The Journal of Physiology 591, no. 17: 4099–4116. 10.1113/jphysiol.2013.256461. 23818691 PMC3779105

[vms371062-bib-0022] Remme, C. A. 2023. “SCN5A Channelopathy: Arrhythmia, Cardiomyopathy, Epilepsy and Beyond.” Philosophical Transactions of the Royal Society B 378, no. 1879: 20220164. 10.1098/rstb.2022.0164. PMC10150216 37122208

[vms371062-bib-0023] Richardson, C. A. , and P. A. Flecknell . 2005. “Anaesthesia and Post‐Operative Analgesia Following Experimental Surgery in Laboratory Rodents: Are We Making Progress?” Alternatives to Laboratory Animals 33, no. 2: 119–127. 10.1177/026119290503300207. 16180987

[vms371062-bib-0024] Saha, J. K. , J. Xia , J. M. Grondin , S. K. Engle , and J. A. Jakubowski . 2005. “Acute Hyperglycemia Induced by Ketamine/Xylazine Anesthesia in Rats: Mechanisms and Implications for Preclinical Models.” Experimental Biology and Medicine 230, no. 10: 777–784. 10.1177/153537020523001012. 16246906

[vms371062-bib-0025] Savić Vujović, K. , A. Jotić , B. Medić , et al. 2023. “Ketamine, an Old‐New Drug: Uses and Abuses.” Pharmaceuticals 17, no. 1: 16. 10.3390/ph17010016. 38276001 PMC10820504

[vms371062-bib-0026] Savio‐Galimberti, E. , M. H. Gollob , and D. Darbar . 2012. “Voltage‐Gated Sodium Channels: Biophysics, Pharmacology, and Related Channelopathies.” Frontiers in Pharmacology 3: 124. 10.3389/fphar.2012.00124. 22798951 PMC3394224

[vms371062-bib-0027] Shiels, H. A. 2022. “Avian Cardiomyocyte Architecture and What It Reveals About the Evolution of the Vertebrate Heart.” Philosophical Transactions of the Royal Society B 377, no. 1864: 20210332. 10.1098/rstb.2021.0332. PMC9527935 36189815

[vms371062-bib-0028] Sleigh, J. , M. Harvey , L. Voss , and B. Denny . 2014. “Ketamine – More Mechanisms of Action Than Just NMDA Blockade.” Trends in Anaesthesia and Critical Care 4, no. 2‐3: 76–81. 10.1016/j.tacc.2014.03.002.

[vms371062-bib-0029] Veerman, C. C. , A. A. Wilde , and E. M. Lodder . 2015. “The Cardiac Sodium Channel Gene SCN5A and Its Gene Product NaV1. 5: Role in Physiology and Pathophysiology.” Gene 573, no. 2: 177–187. 10.1016/j.gene.2015.08.062. 26361848 PMC6636349

[vms371062-bib-0030] Zhang, X.‐L. , T.‐P. Wei , F. Yang , H.‐H. Liu , L.‐L. Qian , and R.‐X. Wang . 2025. “Voltage‐Gated Sodium Channels: A Therapeutic Target in Ischemic Heart Disease.” Reviews in Cardiovascular Medicine 26, no. 6: 27140. 10.31083/RCM27140. 40630435 PMC12230830

[vms371062-bib-0031] Zhou, X. , L. Ren , J. Huang , Y. Zhang , Y. Cai , and J. Pu . 2023. “Novel SCN5A Frame‑Shift Mutation Underlying in Patient With Idiopathic Ventricular Fibrillation Manifested With J Wave in Inferior Lead and Prolonged S‑Wave in Precordial Lead.” Experimental and Therapeutic Medicine 25, no. 6: 287. 10.3892/etm.2023.11986. 37206574 PMC10189605

[vms371062-bib-0032] Zhou, Y. , and J. Zhang . 2023. “Neuronal Activity and Remyelination: New Insights Into the Molecular Mechanisms and Therapeutic Advancements.” Frontiers in Cell and Developmental Biology 11: 1221890. 10.3389/fcell.2023.1221890. 37564376 PMC10410458

